# The 2024 LI-RADS treatment response update: practical reporting after non-radiation and radiation locoregional therapies for hepatocellular carcinoma

**DOI:** 10.1186/s13244-026-02290-9

**Published:** 2026-04-27

**Authors:** Jernej Lučev

**Affiliations:** Department of Radiology, UMC Maribor, Maribor, Slovenia

**Keywords:** LI-RADS, Hepatocellular carcinoma, Treatment response, Stereotactic body radiotherapy, Transarterial chemoembolization

## Abstract

**Abstract:**

This review critically appraises the 2024 Liver Imaging Reporting and Data System (LI-RADS) Treatment Response Algorithm (TRA), which introduces separate non-radiation and radiation treatment response pathways and optional MRI ancillary features, and provides evidence-based guidance for clinical implementation. The updated framework addresses key limitations of the prior algorithm, including moderate sensitivity for residual viable hepatocellular carcinoma (HCC), ambiguity of the Equivocal category, and overcalling after radiation therapy. The 2024 update simplifies viability assessment by emphasizing mass-like enhancement as the dominant sign of residual tumor, introduces a radiation-specific Nonprogressing category based on interval behavior, and allows cautious use of diffusion-weighted and T2-weighted MRI ancillary features in selected non-radiation cases. These changes are expected to improve earlier detection of clinically relevant residual disease while reducing false-positive viable calls after radiation-based therapies. Successful implementation requires high-quality imaging, treatment-specific reporting, multidisciplinary review, and awareness of current scope limitations, particularly in patients receiving systemic or combined therapies.

**Critical relevance statement:**

The LI-RADS v2024 update critically assesses prior flaws by establishing separate non-radiation and radiation treatment response algorithms, directly advancing clinical practice by reducing false-positive viable calls and standardizing surveillance after complex therapies.

**Key Points:**

- Update resolves inconsistency by establishing separate algorithms for non-radiation and radiation therapies, significantly reducing false-positive “Viable” calls after radiation treatment.
- Viability criteria are streamlined to rely on mass-like enhancement and optionally include DWI/T2 ancillary features, improving sensitivity for early recurrence detection. These ancillary features require cautious interpretation to minimize false-positive viable calls.
- Viable lesions prompt intervention, while Nonprogressing lesions warrant close, standardized 3-month surveillance to confirm definitive local control. Typically, short-interval follow-up is performed at ~3 months, but the optimal stability threshold remains undefined.

**Graphical Abstract:**

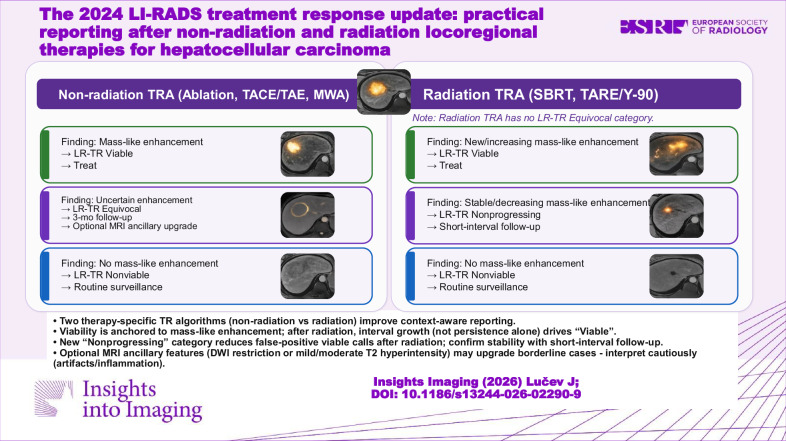

## Introduction

Liver Imaging Reporting and Data System (LI-RADS) is a standardized imaging framework for hepatocellular carcinoma (HCC) that spans surveillance, diagnosis, and post-treatment response assessment [1–3]. The LI-RADS Treatment Response Algorithm (TRA) was introduced in 2017 to guide imaging evaluation of HCC after locoregional therapy [2]. It defines categories conveying the probability of residual viable tumor—from LR-TR Nonviable (no remaining cancer) to LR-TR Viable (high likelihood of remaining cancer), with LR-TR Equivocal (unsure if cancer remains) for indeterminate cases [4, 5]. This system improved consistency in reporting and communication [5, 6]. Challenges like moderate sensitivity and ambiguity of the Equivocal category prompted the 2024 update, introducing separate criteria for non-radiation vs. radiation-based therapies and refined features for viability [4, 7].

This critical review examines the 2024 LI-RADS CT/MRI Treatment Response Algorithm (v2024), now expanded to cover both non-radiation and radiation locoregional therapies, which appraises its evidence base, clinical applications, and implementation challenges. We synthesize validation studies and inter-reader variability data. We discuss practical integration of TRA into clinical workflows, including biomarker correlation, follow-up protocols, modality selection, and tumor board decision-making. Finally, we critically analyze the feasibility of adopting v2024 in diverse settings, addressing obstacles like imaging limitations and subjective features. By contextualizing the TRA’s evolution, we aim to highlight how this update may influence HCC management and identify areas for future refinement.

## Exhaustive summary of the current evidence

### Diagnostic performance and validation

The LI-RADS TRA has been extensively studied since its inception, demonstrating overall high specificity but only moderate sensitivity for detecting residual HCC after therapy. A 2021 meta-analysis (primarily evaluating the 2017 version) reported a pooled sensitivity of approximately 62% and specificity of approximately 87% for identifying residual viable tumor following locoregional treatment [8, 9]. This implies that while LR-TR Viable assessments have strong positive predictive value (few false-positives), a substantial fraction of truly residual tumors may be missed (false-negatives), often due to imaging relying on contrast enhancement as a surrogate for tumor vascularity [10]. For example, “complete response” on imaging (LR-TR Nonviable) does not always equate to complete pathologic necrosis, as tiny foci of tumor can persist without visible enhancement [10]. Thus, a nonviable designation should be interpreted with caution and followed by close surveillance to ensure no tumor regrowth.

Notably, the LI-RADS v2017 criteria required one of three major imaging features—arterial phase hyperenhancement (APHE), washout appearance, or enhancement similar to pretreatment—to declare a lesion LR-TR Viable [10]. Because two of these features were relatively insensitive, the 2024 update simplified the imaging criteria to a single dominant feature, to improve sensitivity—a mass-like enhancement in any phase [3, 4]. Mass-like enhancement refers to any new or residual enhancing tissue that forms a discrete nodule or thick irregular tissue in or around the treated zone [4, 11]. This change was driven by evidence that focusing on the presence of an enhancing nodule can catch recurrences at a smaller size and earlier time points. Indeed, a recent study showed that using the v2024 criterion of “mass-like enhancement (any phase)” enabled detection of HCC recurrence at a median of 5.1 months post-surgery versus 12 months with older criteria, and at a smaller lesion size (approximately 1.5 cm vs. 2.1 cm) [4, 12]. Early data suggest this streamlined approach maintains specificity while substantially improving sensitivity for tumor recurrence [4, 12].

### Inter-reader reliability

Consistency in applying LI-RADS TRA categories among radiologists is critical for widespread adoption. Early studies of the 2017 algorithm showed substantial interobserver agreement overall, with meta-analytic kappa values of approximately 0.70 (70–71% agreement) [9]. The highest agreement was in clear-cut cases of nonviable or obviously viable tumors, whereas intermediate cases saw more reader discrepancy. In particular, the LR-TR Equivocal category had notable variability [13]. Bartnik et al reported only fair-to-moderate agreement on LR-TR Equivocal assignments and found that a significant portion of lesions flagged as Equivocal ultimately harbored residual tumor on follow-up [13]. The v2024 update addresses this by refining how equivocal appearances are handled: for non-radiation treatments, the Equivocal category remains but can be optionally upgraded to Viable if certain ancillary features are present, whereas for radiation treatments, the equivocal category was removed entirely [2, 4]. Overall, inter-reader reliability is expected to improve with clearer criteria (mass-like enhancement) and fewer ambiguous categories. Initial experience with v2024 in a multi-reader study showed higher concordance in identifying recurrence as either Nonviable or Viable, though formal kappa comparisons are pending publication. Training and familiarization with the new definitions will be important to maintain and further improve inter-reader agreement (Tables 1 and 2).

**Table 1:** Summary of major LI-RADS v2024 TRA changes and rationale

Feature	Prior TRA (v2017/2018)	New TRA (v2024)	Rationale/clinical impact
Algorithm core	Single algorithm for all locoregional therapies (LRT).	Separate **Non-Radiation TRA** and **Radiation TRA**.	Accounts for delayed necrosis pattern after SBRT/TARE, **reducing false-positive “Viable” calls** after radiation.
Viability criteria	APHE, Washout, or Pretreatment appearance required.	Single dominant criterion: **Mass-like enhancement** in any phase.	**Improves sensitivity** for detecting early recurrence by simplifying positive findings and catching smaller tumors earlier.
Nonprogressing category	N/A	New category for **Radiation TRA only**.	Allows **observation of stable enhancement** after radiation without automatic LR-TR Viable assignment.
Ancillary features	N/A for TRA (only for Dx).	Optional use of **DWI restriction and T2 hyperintensity** to upgrade Equivocal to Viable (Non-Radiation TRA) or Nonprogressing to Viable (Radiation TRA).	Leverages functional MRI to resolve indeterminate cases and **improve sensitivity**.

**Table 2:** Levels of evidence supporting key statements

Statement (e.g.)	Key citation(s)	Level of evidence (e.g., A: Meta-analysis, B: RCT, C: Retrospective, D: Expert Guidance/Review)
LI-RADS v2017 showed high specificity but moderate sensitivity for residual viable tumor.	[6–8]	A (Systematic review/meta-analysis)
The new Nonprogressing category reduces false-positive Viable calls after SBRT.	[4, 16, 17]	C (Retrospective study/expert consensus)
Optional ancillary features improve sensitivity and address ambiguity in LR-TR Equivocal cases.	[10, 11]	C (Retrospective cohort)
Close follow-up (e.g., 3 months) is advised for equivocal or inconclusive responses.	[21]	D (Expert consensus)
Integrating AFP (Alpha-Fetoprotein) trends with imaging improves post-treatment management.	[18]	D (Practice guidance)

### Validation in non-radiation therapies

Most evidence to date for LI-RADS TRA pertains to locoregional therapies that induce immediate tumor necrosis, such as thermal ablation and transarterial chemoembolization (TACE). In these settings, the algorithm’s performance has been favorable. Multiple retrospective studies have validated that LR-TR Viable on post-treatment MRI strongly predicts true residual disease requiring further therapy, whereas LR-TR Nonviable correlates with pathologic necrosis or sustained local control in the majority of cases [8, 10, 14]. For example, studies have found that the LR-TR criteria after thermal ablation had a high specificity (approximately 95%) for pathologic viable tumor in explants [10]. Chaudhry et al similarly showed that for ablated HCCs, radiologists could reliably categorize treatment success vs. failure using LI-RADS, with few false-positives (specificity more than 90%) and substantial reader agreement [8, 9, 14]. However, these studies also revealed that some patients with LR-TR Nonviable lesions still developed local recurrence months later, reflecting the algorithm’s imperfect sensitivity and the possibility of microscopic disease.

### Need for radiation therapy-specific criteria

A major limitation of the original (2017) TRA was its unproven applicability to radiation-based treatments like transarterial radioembolization (TARE with Y-90) and stereotactic body radiotherapy (SBRT). These therapies cause tumor death via delayed radiation necrosis rather than acute ischemia, often resulting in persistent enhancement and slow regression over time, even if no viable cancer remains [15, 16]. Using the prior criteria, any such residual enhancement was often classified as LR-TR Viable, which was problematic. For example, Mendiratta-Lala et al reported that approximately 45% of SBRT-treated HCCs with residual enhancement on MRI had complete pathological necrosis (no live tumor) in explants [15–17].

Recognizing this, the LI-RADS committee developed a separate Radiation TRA algorithm in 2024 to account for the unique delayed response pattern [4]. Under v2024, when assessing lesions post-TARE or SBRT, a persistently enhancing, stable or decreasing area is now labeled LR-TR Nonprogressing, rather than automatically “viable” [4]. Only if an enhancing focus is new or enlarging over time is it deemed LR-TR Viable in the radiation algorithm [4, 11]. The Nonprogressing category indicates an indolent or regressing post-radiation change, presumed to reflect treatment effect rather than active tumor [4, 18]. The Radiation TRA has no Equivocal category. This bifurcated approach was strongly driven by the evidence gap and clinical need, as the v2017 criteria were overestimating viable tumor after radiation treatments (Table 1) [15, 17]. Early reports suggest that applying the new radiation-specific algorithm substantially reduces false-positive viable calls.

An important unresolved issue is the minimum duration of radiologic stability required before confidently excluding a viable tumor in a lesion categorized as LR-TR Nonprogressing. The v2024 framework emphasizes interval change, but optimal time thresholds (and the number of stable follow-up examinations) are not yet evidence-based and may vary by radiation modality, baseline lesion characteristics, and imaging technique. This represents a key area for future validation studies and should be considered when crafting follow-up recommendations.

### Ancillary features and MRI advances

Another enhancement in LI-RADS v2024 TRA is the incorporation of ancillary MRI features to increase diagnostic confidence [2, 7]. The updated TRA allows two MRI-based ancillary features to optionally “upgrade” a lesion from equivocal/nonprogressing to viable; these two are diffusion restriction and mild-to-moderate T2 hyperintensity [2, 7]. These additions stem from studies indicating that viable HCC tends to show restricted diffusion and T2 hyperintensity due to retained cellularity and edema, whereas fully necrotic tissue does not [3, 12]. For example, a study by Kim et al demonstrated that adding DWI and T2 criteria improved sensitivity for residual HCC from 57% to 71% with minimal loss of specificity [7]. By leveraging MRI’s functional imaging capabilities (DWI, T2), the hope is to capture more subtle residual disease and reduce the number of indeterminate cases.

Practical caution is warranted when applying these optional ancillary features. Diffusion-weighted imaging is susceptible to motion and susceptibility artifacts, and high signal intensity may reflect T2 shine-through rather than true restriction; careful correlation with the ADC map and dynamic contrast patterns is essential. Similarly, mild-to-moderate T2 hyperintensity can be seen with post-treatment edema, hemorrhage, or inflammatory change, potentially reducing specificity in the early post-therapy setting. Because these features are sequence-dependent and reader-dependent, it is recommended to use them only as supportive evidence in borderline cases and explicitly acknowledge residual uncertainty when findings are discordant.

## Detailed evidence-based applications in clinical practice

### Integration with alpha-fetoprotein (AFP) and other biomarkers

Effective HCC surveillance and post-treatment monitoring often combine imaging with laboratory tests, the most established being serum alpha-fetoprotein (AFP). In clinical practice, LI-RADS TRA assessment should be interpreted alongside AFP trends. For example, an observation categorized as LR-TR Nonviable is reassuring, but if the patient’s AFP is steadily rising, the multidisciplinary team should remain cautious. In such a case, earlier or more aggressive follow-up (e.g., repeat MRI in 2–3 months rather than 6 months) or additional imaging like PET-CT may be warranted despite the “nonviable” imaging appearance. This synergy is supported by guidelines—the 2023 AASLD practice guidance, for instance, recommends imaging every 3–6 months after curative treatment and notes that rising tumor markers should prompt further investigation [18, 19]. LI-RADS v2024 itself does not incorporate AFP, but users are encouraged to mention biomarker status in reports and recommendations. Integration of biomarkers with LI-RADS categories allows a more holistic assessment, leveraging the high specificity of imaging with the systemic insight of tumor markers.

### Follow-up imaging protocols and timing

**Fig. 1:**
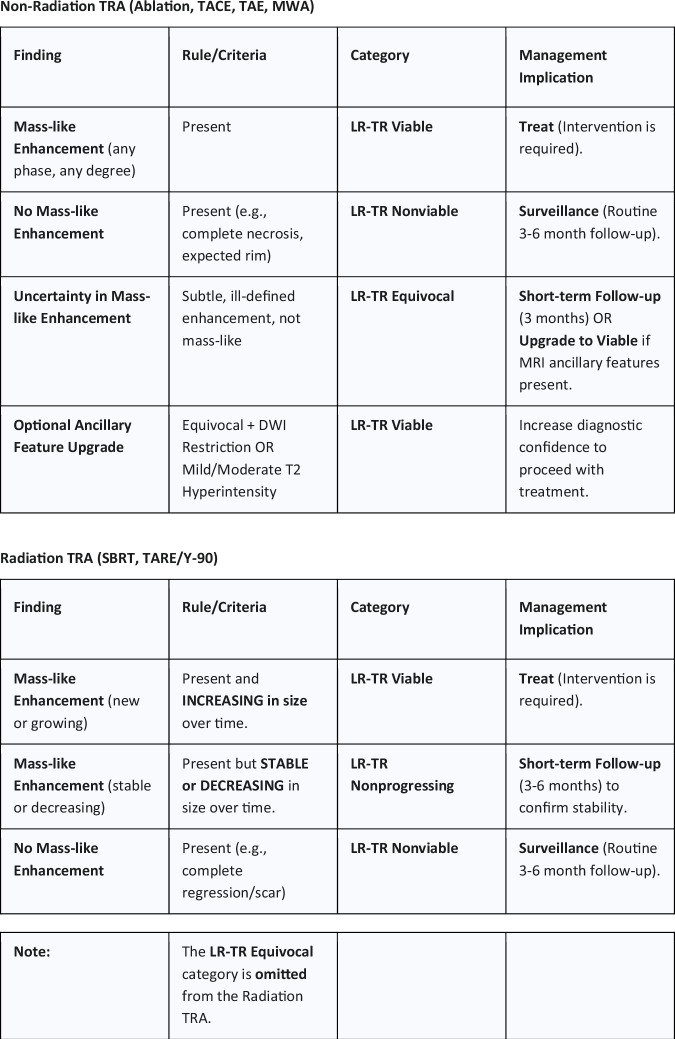
Schematic of non-radiation vs. radiation TRA pathways. This figure outlines the critical decision points and outcomes for post-locoregional therapy assessment in LI-RADS v2024, highlighting the bifurcation between treatment types. The Non-Radiation TRA categorizes any mass-like enhancement as LR-TR Viable, whereas the Radiation TRA categorizes stable or decreasing mass-like enhancement as LR-TR Nonprogressing, acknowledging the delayed nature of the radiation effect

**Fig. 2:**
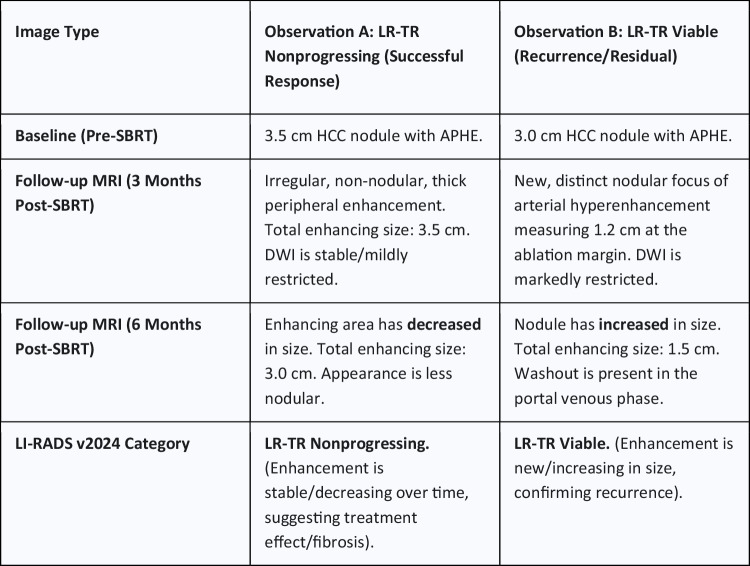
Examples of LR-TR Nonprogressing vs. Viable after SBRT (Conceptual Illustration). This figure illustrates the application of the Radiation TRA based on change over time, comparing an observation with stable/decreasing enhancement (Nonprogressing) against an observation with increasing enhancement (Viable)

A key application of LI-RADS TRA categories is guiding follow-up scheduling and intensity. Standard practice has been to obtain contrast-enhanced imaging at approximately 1-month post-treatment to establish a baseline, then at regular intervals (typically every 3 months for the first year, then spacing to every 4–6 months) if no recurrence is seen (Figs. 1 and 2).

LI-RADS categories refine this approach:

- **LR-TR Viable** findings demand prompt action (confirmatory imaging or direct therapy).
- **LR-TR Nonviable** findings support a return to surveillance.
- The LI-RADS manual recommends 3-month interval imaging for both LR-TR Nonviable and Equivocal cases in the first year [20].
- An equivocal or **Nonprogressing** lesion in the radiation algorithm similarly warrants short-term re-imaging to check for any interval growth. A consensus statement by the ECIO/ESOI specifically suggests that in inconclusive cases regarding residual tumor or recurrence, an additional follow-up at 3 months is advised [21].

**Fig. 3:**
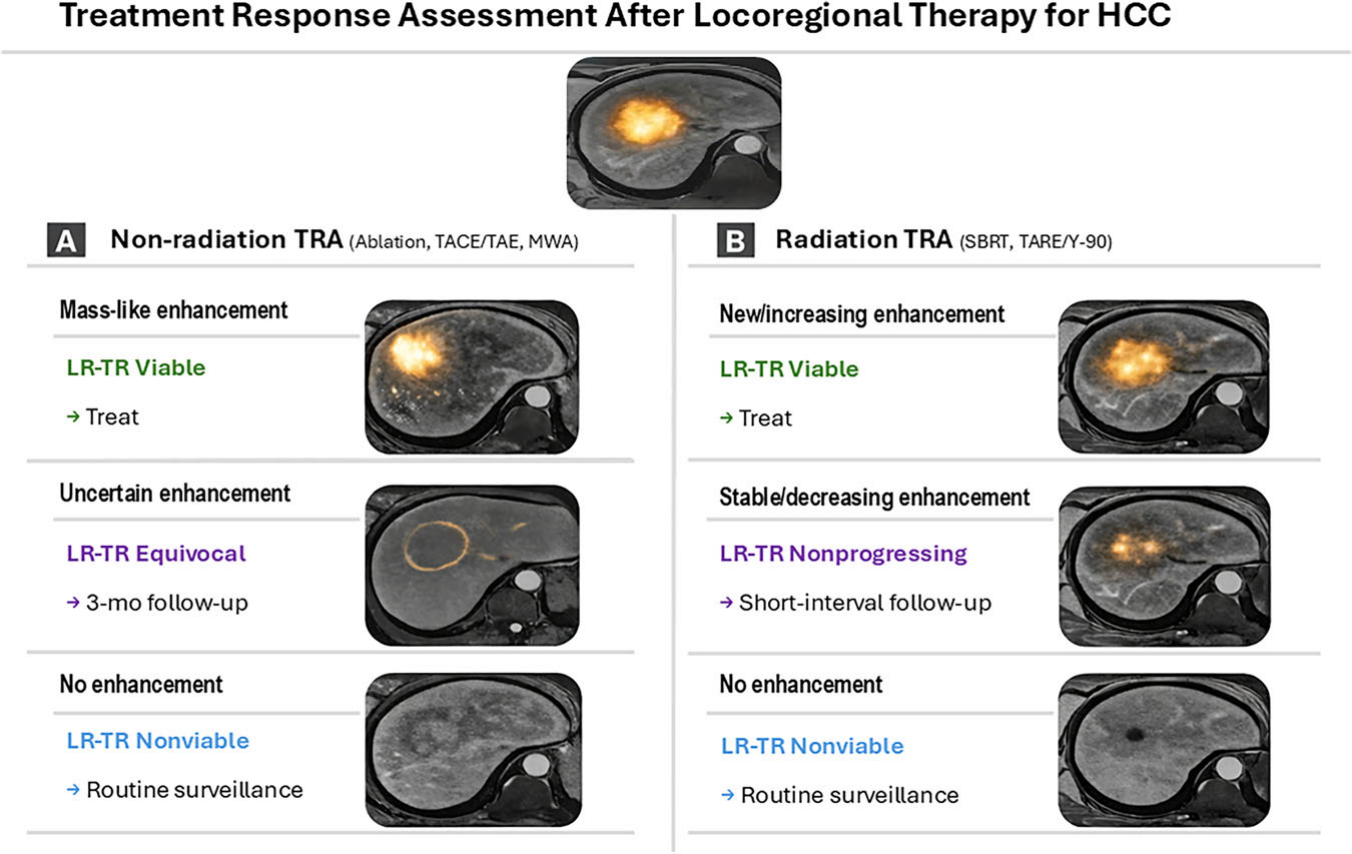
Practical overview of LI-RADS v2024 treatment response assessment after locoregional therapy for HCC. This schematic separates non-radiation therapies - panel **A** (ablation, TACE/TAE, MWA) from radiation therapies - panel **B** (SBRT, TARE/Y-90). For non-radiation therapies, mass-like enhancement indicates LR-TR Viable, uncertain subtle enhancement indicates LR-TR Equivocal, and no enhancement indicates LR-TR Nonviable. For radiation therapies, new or increasing mass-like enhancement indicates LR-TR Viable, stable or decreasing enhancement indicates LR-TR Nonprogressing, and no enhancement indicates LR-TR Nonviable. The figure also summarizes the corresponding management implications: treat for Viable, short-interval follow-up for Equivocal or Nonprogressing, and routine surveillance for Nonviable

A practical approach is: Viable—treat (with necessary pretreatment confirmation), Equivocal or Nonprogressing—re-scan in 3 months, Nonviable—routine surveillance (3-month intervals in year 1, then extending) [20, 21]. The v2024 update’s Nonprogressing category for radiation therapy acknowledges that some lesions can be observed with follow-up rather than immediately treated. Follow-up must also include monitoring the untreated liver for new lesions, applying the LI-RADS diagnostic algorithm in parallel. These treatment-specific pathways and their typical management implications are summarized in Fig. 3.

In practice, short-interval follow-up is commonly performed at approximately 3 months; however, the optimal interval and the total duration of demonstrated stability needed to confirm durable local control after radiation remain uncertain. Centers should integrate imaging with clinical context (e.g., AFP trends) and multidisciplinary discussion, and may favor repeated short-interval assessments when uncertainty persists.

Illustrative case—non-radiation therapy (TACE): After chemoembolization of a 2–3 cm HCC, follow-up MRI shows subtle, ill-defined enhancement without a discrete mass-like component, which would be categorized as LR-TR Equivocal. On the same examination, a focal area demonstrates diffusion restriction with low ADC and mild-to-moderate T2 hyperintensity; in the absence of confounding hemorrhage or inflammation, v2024 allows optional upgrade to LR-TR Viable, prompting confirmatory imaging and/or retreatment as agreed in the multidisciplinary tumor board.

Illustrative case—radiation therapy (SBRT/TARE): At 3-month MRI after SBRT or Y-90 radioembolization, a treated lesion shows persistent mass-like enhancement that is stable in size compared with baseline and is categorized as LR-TR Nonprogressing, supporting short-interval re-imaging rather than immediate retreatment. On a subsequent examination, the enhancing focus enlarges, meeting interval growth criteria and is reclassified as LR-TR Viable, triggering salvage therapy consideration.

### Imaging modality selection

The v2024 TRA update is specified for CT and MRI, which are the workhorses for HCC imaging.

- **MRI:** Multiphasic contrast-enhanced MRI is generally preferred for its superior sensitivity in detecting small foci of residual tumor—thanks to higher contrast resolution and the addition of diffusion-weighted imaging and hepatobiliary phase (when gadoxetate is used) [21, 22]. MRI can better characterize indeterminate enhancement and leverage ancillary features to identify viability [12].
- **CT:** CT remains widely used due to availability, speed, and lower cost. CT is particularly useful after conventional Lipiodol TACE: radiopaque lipiodol deposition within the tumor acts as a marker of necrosis on a non-contrast CT [21].
- **Contrast-enhanced ultrasound (CEUS):** CEUS is now formally recognized with its own LI-RADS CEUS TRA algorithm (introduced concurrently in 2024) [2]. CEUS can provide real-time evaluation of lesion perfusion, especially helpful for subcentimeter tumors or in patients who cannot undergo CT/MRI [2, 23].

The v2024 TRA does not dictate modality, but its developers emphasize using the best available technique in each case [21]. MRI with extracellular contrast or gadoxetate is often the first choice for its sensitivity and ability to apply the full LI-RADS criteria.

### Workflow in multidisciplinary tumor boards

Management of HCC is typically determined by a multidisciplinary tumor board (MTB). The LI-RADS TRA categories serve as a common language in these meetings, facilitating clear communication.

**Fig. 4:**
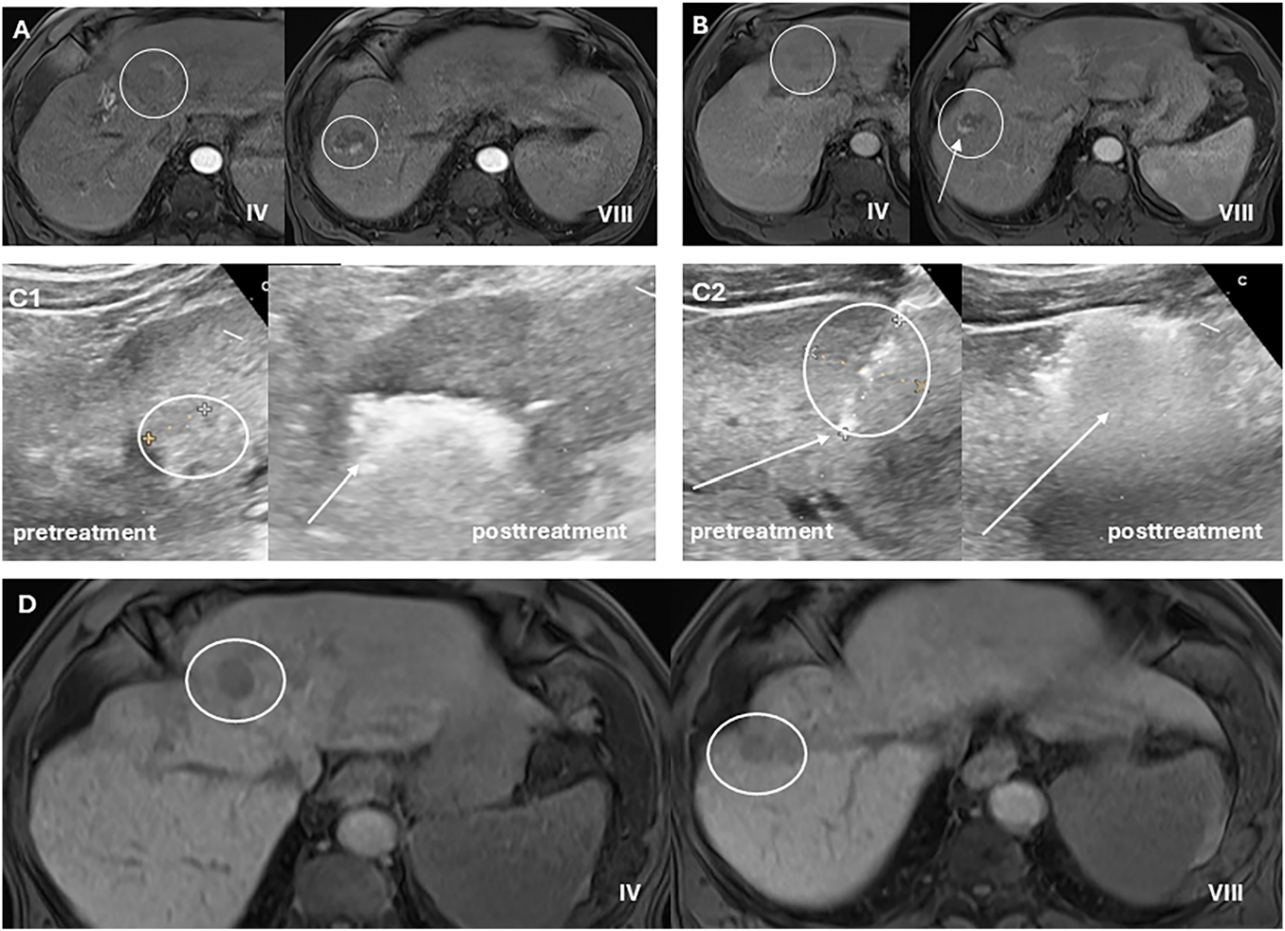
Illustrative non-radiation treatment response case with sequential retreatment. A 68-year-old man with Child-Pugh A cirrhosis and chronic portal vein thrombosis with cavernous transformation had histologically confirmed HCC in segment 8, with a small additional lesion in segment 4A. He underwent DEB-TACE of the segment 8 tumor in June 2025; the segment 4 lesion was not clearly differentiated angiographically at that time. **A** Baseline gadoxetic acid-enhanced MRI demonstrates the segment 4 and segment 8 observations (both circled). **B** Follow-up MRI after DEB-TACE shows interval increase of the segment 4 lesion (circled) and residual nodular enhancement within the treated segment 8 lesion (arrow), consistent with persistent viable disease. **C1** Intraprocedural ultrasound before and after microwave ablation of the segment 4 lesion: the pretreatment lesion is circled, and the post-treatment image shows gas-related ablation artifact (arrow). **C2** Intraprocedural ultrasound before and after microwave ablation of the segment 8 lesion: the pretreatment lesion is circled, the tip of the microwave ablation antenna is indicated by the arrow, and the post-treatment image shows gas-related change (arrow). **D** Follow-up MRI after microwave ablation demonstrates no residual mass-like enhancement in either the segment 4 or segment 8 treatment zone, consistent with complete local response after sequential non-radiation therapy
- For LR-TR Equivocal or Nonprogressing lesions, the MTB often opts for conservative management with close follow-up imaging, rather than immediate treatment.
- A reported LR-TR Nonprogressing (in a radiation case) signals stability and likely effective treatment, which might translate to “ongoing observation” rather than declaring treatment failure, a critical change from v2017.

- For LR-TR Viable lesions, there is consensus that active treatment is needed. The earlier detection of recurrence with v2024 (at smaller sizes) may improve eligibility for salvage therapies [12]. As an illustrative non-radiation treatment response case, we present a 68-year-old man with Child-Pugh A cirrhosis, chronic portal vein thrombosis with cavernous transformation, and histologically confirmed HCC in segment 8, with a small additional lesion in segment 4A. He underwent DEB-TACE of the segment 8 tumor in June 2025; the segment 4A lesion was not clearly differentiated angiographically at that time. Follow-up gadoxetic acid-enhanced MRI showed an interval increase of the segment 4 lesion to 18 mm and residual nodular enhancement within the treated segment 8 lesion, consistent with persistent viable disease in both sites. Both lesions were subsequently identified on ultrasound and treated with percutaneous microwave ablation. On follow-up MRI after ablation, no residual mass-like enhancement was identified in either treatment zone, illustrating how multimodality imaging can support sequential retreatment decisions and confirm complete local response after non-radiation therapy (Fig. 4).

The standardized reporting system (LI-RADS) ensures all MTB members and referring clinicians understand the report the same way, reducing ambiguity and promoting evidence-based decision-making.

## Considerations on feasibility and obstacles in the implementation of the technique in clinical practice

### Modality limitations and resource constraints

A significant implementation challenge is ensuring access to recommended imaging modalities (MRI in particular) at appropriate time points. The v2024 algorithm places emphasis on MRI ancillary features, subtly favoring MRI’s capabilities [12, 22]. However, not all centers have the capacity to perform serial liver MRIs at 3-month intervals for every post-treatment patient. Reliance on CT might reduce the full benefit of v2024.

Another aspect is scheduling and timing. The algorithm assumes imaging “not too soon” after treatment (approximately 4–6 weeks post-therapy for a baseline scan) [4, 5]. If follow-up is delayed due to backlogs, some early recurrences might grow beyond the “small size” advantage that v2024 is meant to catch [12].

### Subjectivity of imaging features

Despite clearer criteria, some inherent subjectivity remains in interpreting post-treatment imaging. What exactly constitutes “mass-like enhancement” can be debatable (e.g., is a faint, ill-defined region of enhancement a mass or just inflammatory blush?). The optional use of ancillary features introduces another level of discretion. One radiologist may upgrade an equivocal lesion based on perceived mild diffusion restriction, whereas another might not be convinced the signal is meaningful.

Beyond enhancement patterns, the optional MRI ancillary features introduce additional interpretive variability. DWI signal depends on acquisition parameters (e.g., b-values, fat suppression, field strength) and may be degraded by motion; T2 shine-through can mimic restriction unless ADC is carefully reviewed. Likewise, T2 hyperintensity can be nonspecific in the early post-treatment liver, where edema, hemorrhage, and reactive hyperemia may overlap with viable tumor. These pitfalls reinforce that ancillary features should be applied conservatively and documented transparently in the report narrative.

The new category, Nonprogressing, in the radiation algorithm relies on comparing lesion size over time. Measurement variability on MRI/CT can be significant, and distinguishing truly “stable” from a slight increase can be challenging. This subjectivity means that in less experienced hands, the categories might be applied inconsistently.

### Reproducibility and training across settings

Ensuring that the updated TRA can be reproduced with the same effectiveness outside of academic centers is a major challenge. Community radiologists or those in low-volume centers may not be as familiar with LI-RADS categories. A key issue is recognizing when to use the radiation algorithm (i.e., knowing the treatment type), which requires complete and accurate treatment histories from referring clinicians. If treatment information is incomplete (e.g., TACE vs. Y-90), misclassification is possible.

The perceived complexity of LI-RADS has been a barrier for some [1]. The v2024 update, by adding algorithms, arguably makes it more complex at first glance, requiring radiologists to choose between two TRA pathways. Standardizing imaging protocols is necessary to reliably apply TRA, as inconsistent scan quality can render a study non-evaluable. Continuing medical education and perhaps automated decision-support tools may help to improve reproducibility.

### Verification and outcomes tracking

Implementing LI-RADS TRA should ideally go hand-in-hand with outcomes tracking to confirm its efficacy. Few general centers have the infrastructure for this continuous quality improvement. Early indications are that v2024 TRA has led to a stage migration, indicating that more lesions are being called viable earlier and fewer are left in limbo as equivocal.

Finally, LI-RADS explicitly does not cover tumors treated with systemic agents (e.g., immunotherapy, tyrosine kinase inhibitors) [10, 24]. As systemic therapy for HCC becomes more common, the lack of a standardized imaging response system is a major gap. In these cases, radiologists must revert to RECIST or mRECIST criteria, which adds complexity to interpreting combination therapies [24, 25]. These complex scenarios are currently managed by expert opinion rather than an algorithm, an acknowledged limitation of LI-RADS [1, 4].

### Summary statement/main recommendations (3 bullet points)

- Apply the correct core algorithm (non-radiation or radiation) based on treatment type; use a single dominant feature, mass-like enhancement, as the key sign of viability, which improves sensitivity for early recurrence detection.
- Use MRI, when feasible, for equivocal cases, and judiciously apply the diffusion restriction and T2 hyperintensity ancillary features to upgrade low-suspicion lesions, particularly when imaging findings and AFP trends are discordant. Be mindful of artifacts and early post-treatment inflammatory change that can mimic these features.
- Guide management by stratifying urgency: Viable lesions prompt intervention; Equivocal/Nonprogressing lesions warrant close surveillance with short-interval (approx. 3-month) follow-up imaging to confirm stability or progression, as discussed in a multidisciplinary setting. The optimal duration of stability needed to confirm durable local control after radiation remains an important area for future study.

## Supplementary information

ELECTRONIC SUPPLEMENTARY MATERIAL

## Data Availability

The minimal dataset supporting the results of this article—specifically the curated list of literature, key findings, and assessed levels of evidence—is provided in the Electronic Supplementary Material (ESM_Evidence_Matrix.xlsx), which is available in an editable format alongside this submission.

## Abbreviations

AFP: Alpha-fetoprotein
APHE: Arterial phase hyperenhancement
CEUS: Contrast-enhanced ultrasound
HCC: Hepatocellular carcinoma
LI-RADS: Liver Imaging Reporting and Data System
SBRT: Stereotactic body radiotherapy
TACE: Transarterial chemoembolization
TARE: Transarterial radioembolization
TRA: Treatment response algorithm
