# Supplementary material for: The 2024 LI-RADS treatment response update: practical reporting after non-radiation and radiation locoregional therapies for hepatocellular carcinoma
Source: Insights Imaging. 2026 Apr 27;17:121. doi: 10.1186/s13244-026-02290-9 (PMC13121681; doi:10.1186/s13244-026-02290-9)
Supplement: Supplementary file 1 — ELECTRONIC SUPPLEMENTARY MATERIAL [file 13244_2026_2290_MOESM1_ESM.pdf]

The 2024 LI-RADS Treatment Response update: practical reporting after non-radiation and radiation locoregional therapies for hepatocellular carcinoma

ELECTRONIC SUPPLEMENTARY MATERIAL

This matrix provides a detailed summary of the evidence cited throughout the critical review manuscript, categorized by the type of study and level of evidence.

Citation #	First Author	Year	Journal	DOI	Key Finding Supported	Study Type	Level of Evidence (A-D)
[1]	Chernyak V.	2023	Radiology	10.1148/radiol.222801	Overview of LI-RADS evolution and future directions.	Narrative Review	D
[2]	Choi SH	2024	Korean J Radiol	10.3348/kjr.2024.0161	Overview of the 2024 updates, especially Non-Radiation TRA, Radiation TRA, and ancillary features.	Review	D
[3]	Elsayes KM	2019	J Hepatocell Carcinoma	10.2147/JHC.S186239	Conceptual and historical review of LI-RADS and its integration into AASLD guidance.	Review	D
[4]	Aslam A	2024	Radiology	10.1148/radiol.232408	Summary of the v2024 TRA updates, including	Consensus Statement/Update	D

					mass-like enhancement and the bifurcated algorithm.		
[5]	Shropshire EL	2019	Radiology	10.1148/radiol.2019182135	Performance and diagnostic accuracy of the TRA (v2017/2018).	Retrospective Cohort	C
[6]	Kim TH	2021	Abdom Radiol (NY)	10.1007/s00261-021-03122-8	Sensitivity and specificity of the v2017 TRA.	Systematic Review and Meta-analysis	A
[7]	Gupta P	2021	Eur Radiol	10.1007/s00330-021-07837-6	Diagnostic accuracy of the LI-RADS locoregional treatment response criteria.	Systematic Review and Meta-analysis	A
[8]	Kim DW	2021	Diagnostics (Basel)	10.3390/diagnostics11020237	Interreader reliability of the treatment response algorithm.	Systematic Review and Meta-analysis	A
[9]	Martin M	2023	Hepatoma Res	10.20517/2394-5079.2022.95	Evidence-based update of the LI-RADS treatment response algorithm after liver-directed therapy.	Review	D
[10]	Park S	2020	Radiology	10.1148/radiol.2020192797	Diagnostic performance and	Retrospective Comparative Study	C

					value of ancillary MRI features compared with enhancement patterns at CT and MRI.		
[11]	Kim YY	2022	AJR Am J Roentgenol	10.2214/AJR.21.26677	Impact of ancillary MRI features on TRA diagnostic performance after locoregional treatment.	Retrospective Cohort	C
[12]	Bartnik K	2022	Abdom Radiol (NY)	10.1007/s00261-021-03272-9	Inter-observer agreement using the v2018 CT TRA in patients treated with conventional TACE.	Retrospective Cohort	C
[13]	Chaudhry M	2020	Radiology	10.1148/radiol.2019191581	Evaluation of ablated HCC with the v2018 MRI TRA; equivocal findings may still indicate viable tumor.	Retrospective Cohort	C
[14]	Jiao Y	2022	Health Phys	10.1097/HP.0000000000001601	Radiation-induced cell death mechanisms relevant to the	Review	D

					biological basis of Radiation TRA.		
[15]	Kielar A	2018	Abdom Radiol (NY)	10.1007/s00261-017-1281-6	Locoregional therapies and the original TRA algorithm, including early interpretive caveats.	Review	D
[16]	Mendiratta-Lala M	2022	Int J Radiat Oncol Biol Phys	10.1016/j.ijrobp.2021.10.006	Radiologic-pathologic correlation after SBRT demonstrating limitations of the earlier TRA in the radiation setting.	Retrospective Study	C
[17]	Wei H	2025	Eur Radiol	10.1007/s00330-025-11659-1	Temporal evolution after selective internal radiation therapy and support for the Nonprogressing concept.	Retrospective Cohort	C
[18]	Singal AG	2023	Hepatology	10.1097/HEP.0000000000000466	AASLD practice guidance on HCC, including surveillance, diagnosis, and treatment follow-up.	Practice Guidance	D

[19]	Marrero JA	2018	Hepatology	10.1002/hep.29913	AASLD practice guidance on HCC diagnostic criteria, staging, and management.	Practice Guidance	D
[20]	Voizard N	2019	Insights Imaging	10.1186/s13244-019-0801-z	Pictorial review of treatment response assessment principles with LI-RADS.	Pictorial Review	D
[21]	Maas M	2020	Insights Imaging	10.1186/s13244-020-00884-5	ECIO-ESOI evidence- and consensus-based recommendations on post-intervention follow-up timing and modality.	Expert Consensus	D
[22]	De Muzio F	2022	Diagnostics (Basel)	10.3390/diagnostics12071655	Narrative review of LI-RADS in liver tumors, including modality considerations and pitfalls.	Narrative Review	D
[23]	Wilson SR	2018	Abdom Radiol (NY)	10.1007/s00261-017-1250-0	CEUS LI-RADS algorithm, implementation, and key differences from CT/MRI LI-RADS.	Review	D
[24]	Lencioni R	2010	Semin Liver Dis	10.1055/s-0030-	Modified RECIST	Review	D

				1247132	(mRECIST) assessment for hepatocellular carcinoma.		
[25]	Faggian G	2026	Italian Journal of Medicine	10.4081/itjm.2026.2413	General review of LI-RADS current status and future perspectives in HCC diagnosis.	Review	D
